# Identification of Cellular Factors Required for SARS-CoV-2 Replication

**DOI:** 10.3390/cells10113159

**Published:** 2021-11-13

**Authors:** Aleksandra Synowiec, Malwina Jedrysik, Wojciech Branicki, Adrianna Klajmon, Jing Lei, Katarzyna Owczarek, Chen Suo, Artur Szczepanski, Jingru Wang, Pengyan Zhang, Pawel P. Labaj, Krzysztof Pyrc

**Affiliations:** 1 ViroGenetics—BSL3 Laboratory of Virology, Malopolska Centre of Biotechnology, Jagiellonian University, Gronostajowa 7a, 30-387 Krakow, Poland; a.synowiec@doctoral.uj.edu.pl (A.S.); malwina.jedrysik@gmail.com (M.J.); k.owczarek@uj.edu.pl (K.O.); artur.szczepanski@uj.edu.pl (A.S.); 2 Human Genome Variation Research Group, Malopolska Centre of Biotechnology, Jagiellonian University, Gronostajowa 7a, 30-387 Krakow, Poland; wojciech.branicki@uj.edu.pl (W.B.); adrianna.klajmon@uj.edu.pl (A.K.); 3 Key Laboratory of Public Health Safety, Department of Epidemiology & Ministry of Education, School of Public Health, Fudan University, Shanghai 200032, China; 20211020009@fudan.edu.cn (J.L.); iamsuochen@gmail.com (C.S.); 20211020024@fudan.edu.cn (J.W.); 20211020035@fudan.edu.cn (P.Z.); 4 Taizhou Institute of Health Sciences, Fudan University, Taizhou 225316, China; 5 Microbiology Department, Faculty of Biochemistry, Biophysics and Biotechnology, Jagiellonian University, Gronostajowa 7, 30-387 Krakow, Poland; 6 Bioinformatics Research Group, Malopolska Centre of Biotechnology, Jagiellonian University, Gronostajowa 7a, 30-387 Krakow, Poland

**Keywords:** SARS-CoV-2, coronavirus, CRISPR-Cas9, cellular factors, mechanisms of infection, viral pathogenesis

## Abstract

Severe acute respiratory syndrome coronavirus 2 (SARS-CoV-2) is the recently emerged virus responsible for the COVID-19 pandemic. Clinical presentation can range from asymptomatic disease and mild respiratory tract infection to severe disease with lung injury, multiorgan failure, and death. SARS-CoV-2 is the third animal coronavirus to emerge in humans in the 21st century, and coronaviruses appear to possess a unique ability to cross borders between species and infect a wide range of organisms. This is somewhat surprising as, except for the requirement of host cell receptors, cell–pathogen interactions are usually species-specific. Insights into these host–virus interactions will provide a deeper understanding of the process of SARS-CoV-2 infection and provide a means for the design and development of antiviral agents. In this study, we describe a complex analysis of SARS-CoV-2 infection using a genome-wide CRISPR-Cas9 knock-out system in HeLa cells overexpressing entry receptor angiotensin-converting enzyme 2 (ACE2). This platform allows for the identification of factors required for viral replication. This study was designed to include a high number of replicates (48 replicates; 16 biological repeats with 3 technical replicates each) to prevent data instability, remove sources of bias, and allow multifactorial bioinformatic analyses in order to study the resulting interaction network. The results obtained provide an interesting insight into the replication mechanisms of SARS-CoV-2.

## 1. Introduction

The first coronaviruses, so named for their unique crown of spike (S) proteins, were discovered in animals by electron microscopy approximately 80 years ago. Four seasonal coronaviruses associated with relatively mild respiratory tract disease have been identified (HCoV-NL63, HCoV-OC43, HCoV-HKU1, and HCoV-229E). These species emerged in humans by zoonotic transfer, with HCoV-NL63 identified as the oldest human coronavirus by molecular clock analyses, entering the human population approximately 1000 years ago [1]. HCoV-HKU1 is the youngest of these seasonal coronaviruses and is thought to have crossed the species barrier in the 20th century [2]. Some speculate that, at the time of emergence, these species caused severe disease in immunologically naïve humans [3].

The emergence of SARS-CoV and MERS-CoV in humans has shown that these viruses have a unique talent to cross borders between species and that there is a rich reservoir of coronaviruses in animals [4]. However, little action has been taken to prepare for the emergence of coronaviruses in humans, and, consequently, we were unprepared for the emergence of SARS-CoV-2 in 2019. Considering the evolutionary history of human coronaviruses and the fact that in the 21st century, three zoonotic transmission events already took place, one may say that these viruses show a peculiar ability to tune up to the cellular machinery of different species. Considering the current threat from SARS-CoV-2, and the risk that other coronaviruses may cross from their animal reservoir into humans, understanding this broad-range fitness is essential.

CRISPR/Cas9 screening platforms are versatile tools that enable us to map virus-cell interaction networks. In the long-term perspective, this knowledge may lead to a better understanding of the underlying mechanism of pathogenesis and the development of personalized disease management [5,6]. Originating from the bacterial adaptive-immunity system, CRISPR/Cas9 can perform homology-dependent recognition and cleavage of foreign nucleic acids, guided by short RNA sequences (guide RNAs; gRNAs). In mammalian cells, cleavage of genomic DNA by CRISPR/Cas9 leads to the recruitment of DNA repair machinery and the introduction of indel mutations [7]. gRNAs can be designed so that these indel mutations result in protein truncations [7,8]. The employment of semi-random or broad-spectrum gRNA libraries allows for the inactivation of several genes in a cell population and high-throughput analysis of cellular pathways, involved in the response to viral infection. This approach has already been used for the identification of the vast array of host factors involved in the pathogenesis of many viruses: coronaviruses [9,10,11,12,13], ZIKV [14], HIV-1 [15], or Influenza A virus (IAV) [16]. Direct, point mutagenesis, and a reproducible, relatively easy-to-use CRISPR/Cas9 knock-out screening, make it a powerful tool for the de novo investigation of the virus–host interactome. However, analyses of these high-throughput data have limitations: firstly, each cell may contain several gRNAs and therefore the appearance of false positives in many individual samples is observed; secondly, transduction efficacy can be limited, and some gRNAs may never be transfected into the cell population; finally, the effect of an indel mutation on cell metabolism may impact the abundance of other gRNAs in the cell population. Using the CRISPR/Cas9 screening, the cells that survived the infection with the virus are selected and clonally expanded. Consequently, factors essential for the late stages of the replication cycle will not be identified, as the cell is already irreversibly damaged by robust viral replication. However, one may speculate that if the virus infection is abortive, the cells may survive, and the factors inhibiting entry and replication may be also identified.

Nevertheless, understanding the results also requires a profound knowledge of the virus replication cycle. SARS-CoV-2 enters the cell by virus–cell fusion after priming of the spike protein by cellular proteases. The viral genomic RNA serves in the cytoplasm as a template for the cellular machinery during the production of the non-structural proteins (1a/1ab), responsible for, e.g., the reorganization of intracellular architecture, modulation of innate immune responses, and replication of the viral RNA. In the second stage, the viral replication complex produces a set of sub-genomic mRNAs, which yield structural proteins. Consequently, new viral particles assemble and are transported outside the cell, which eventually dies [17,18].

Here, we describe the complex analysis of the SARS-CoV-2 virus interactome in human cell populations. This analysis was carried out using a HeLa cell line overexpressing the ACE2 receptor on its surface (HeLa^ACE2^), one of the accepted models for studying SARS-CoV-2 pathogenesis [19]. The applied study design incorporates nested replicates to minimize biases originating from the experimental system and, as a result, 48 replicate samples were tested, allowing for comprehensive statistical analysis and comparison with datasets from other studies. The developed analysis pipeline allows for an unbiased assessment of the cellular factors required for the productive SARS-CoV-2 infection and may be used in the future for other pathogens.

## 2. Materials and Methods

### 2.1. Cell Culture

HeLa (ATCC CCL-2), HeLa cells overexpressing ACE2 (HeLa^ACE2^), A549 (ATCC CCL-185), A549 cells overexpressing both ACE2 and TMPRSS2 (A549^ACE2/TMPRSS2^), Vero E6 cells (*Cercopithecus aethiops*; kidney epithelial; ATCC CRL2691586), and HEK293T cells (ATCC CRL-3216; human embryonic kidney) were maintained in Dulbecco’s modified Eagle’s medium (DMEM, high glucose; Thermo Fisher Scientific, Warsaw, Poland) supplemented with 5% heat-inactivated fetal bovine serum (5% DMEM; Thermo Fisher Scientific), penicillin (100 U/mL; Thermo Fisher Scientific), and streptomycin (100 μg/mL; Thermo Fisher Scientific). HeLa^ACE2^ cells were additionally supplemented with G418 (500 μg/mL, Sigma-Aldrich, Poznań, Poland) to maintain the ACE2^+^ population. A549^ACE2/TMPRSS2^ cells were supplemented with blasticidin S (10 μg/mL, Sigma-Aldrich), and puromycin (0.5 μg/mL, Sigma-Aldrich) to maintain the ACE2^+^TMPRSS2^+^ population. Cells were cultured at 37 °C, 5% CO_2_, and 95% humidity. All cell lines were tested every two weeks for mycoplasma contamination either by in-house DAPI staining or LookOut^®^ Mycoplasma PCR Detection Kit (Sigma-Aldrich).

### 2.2. Virus

SARS-CoV-2 (isolate 026V-03883; kindly granted by Christian Drosten, Charité—Universitätsmedizin Berlin, Germany by the European Virus Archive—Global (EVAg)) and mock-infected samples were generated by infecting confluent monolayers of Vero E6 cells at 400 TCID_50_/mL. Seventy-two hours post-infection, virus-containing supernatants were collected, aliquoted, and stored at −80 °C. Infectious samples were titrated as previously described [20,21]. All viral work was performed in a Biosafety Level 3+ laboratory (BSL3+).

### 2.3. GeCKO Plasmids Library Preparation

The human GeCKOv2 CRISPR knock-out pooled library was a gift from Feng Zhang (Addgene #1000000048) and was prepared in Lucigen Endura cells (cat #60242), according to the previously described protocol [22].

### 2.4. Lentiviral Particle Production

Lentiviral vectors were produced by co-transfection of HEK293T cells using the polyethyleneimine (PEI) transfection method. Briefly, 2 × 10^6^ HEK293T cells were seeded on a 10 cm Petri dish (TPP Techno Plastic Products AG, Trasadingen, Switzerland) previously coated with bovine collagen type I (40 µg/mL, Advanced BioMatrix, Carlsbad, CA, USA). Twenty-four hours later, cells were co-transfected with 500 µL of transfection mix: 27 µg of PEI (Sigma-Aldrich), 9 μg GeCKO library (lentiCRISPRv2 one vector system, Library A + Library B), 6.75 μg packaging plasmid psPAX2 (a gift from Didier Trono; Addgene plasmid #12260), and 2.25 μg envelope plasmid pMD2.G (a gift from Didier Trono; Addgene plasmid #12259) diluted in OptiMEM (Thermo Fisher Scientific). A total of 9 μg of pLeGO-G2 (a gift from Boris Fehse; Addgene plasmid #25917), instead of GeCKO library plasmids, was used as a transfection and transduction control. The medium was refreshed after 16 h, and recombinant lentiviruses were harvested 24 h, 48 h, and 72 h later. The supernatant containing vector particles was centrifuged at 500× *g* for 5 min at 4 °C, filtered through 0.22 µm PVDF membrane filters, and concentrated 20 times using Amicon Ultra-0.5 Centrifugal Filter Unit Ultracel 100 kDa cut-off (Sigma-Aldrich). To maintain representative populations of gRNAs, lentiviruses were used directly following preparation.

### 2.5. Transduction, Selection, and Genome-Wide Screening

HeLa^ACE2^ cells were plated on 10 cm plates (TPP Techno Plastic Products AG) at 2 × 10^6^ cells/plate 24 h before transduction. Cells were overlaid with a 5 mL concentrated vector supplemented with polybrene (5 μg/mL, Sigma-Aldrich) for 16 h before the medium was refreshed. After 72 h, cells were passaged into a fresh medium supplemented with puromycin (0.5 μg/mL, BioShop Burlington, Canada) and cultured for 2–3 weeks. Transfected cells (1 × 10^7^) were seeded in the T75 flasks for 24 h before being infected with 5 mL concentrated SARS-CoV-2 virus stock. Fresh medium (2 hpi, 5 mL) was added, and cultures were maintained for 7–10 days at 37 °C, with the medium exchange every 2 days to discard cell debris. The surviving cells were collected and centrifuged, and the genomic DNA was isolated.

### 2.6. Generation of HeLa^ACE2^ Cells

HeLa cells expressing the ACE2 protein (HeLa^ACE2^) were generated using retroviral vectors (Moloney murine leukemia virus system). Retroviruses were prepared using Phoenix-AMPHO cells (ATCC CRL-3213). Briefly, cells were transfected with a pLNCX2 vector (Takara Bio, Mountain View, CA, USA) encoding the ACE2 protein. A total of 27 µg of PEI (Sigma-Aldrich) and 9 µg of the vector, diluted in 500 µL of OptiMEM (Thermo Fisher Scientific) was added to the cells dropwise. At 16 h post-transfection, the medium was refreshed, and the cells were cultured for 48 h at 37 °C. Subsequently, the vector-containing supernatants were harvested 48 h and 72 h after transfection, then aliquoted an d stored at −80 °C. HeLa cells (ATCC CCL-2) cells were cultured in six-well plates (TPP, Trasadingen, Switzerland) and infected with 1 ml of generated retroviruses in the presence of polybrene (5 μg/mL; Sigma-Aldrich). After 24 h of incubation at 37 °C, the cells were cultured in a medium supplemented with G418 (5 mg/ml; BioShop, Burlington, ON, Canada) and passaged for 2 weeks at 37 °C, and the clonal selection was performed afterward. Cells were analyzed as described below.

### 2.7. Generation of A549^ACE2/TMPRSS2^ Cells

Lentiviruses were prepared as described in Section 2.4. Briefly, HEK293T cells were transfected with the pLKO.1-TRC-ACE2 plasmid (based on the Addgene plasmid #10878) and pLEX307-TMPRSS2-blast (Addgene plasmid #158458). To obtain an A549^ACE2/TMPRSS2^ cell line, we co-transduced A549 (ATCC CCL-185™) cells with lentiviral vectors harboring sequences for TMPRSS2 and ACE2. The medium was refreshed after 16 h and passaged 72 h later in fresh medium supplemented with puromycin (0.5 μg/mL, BioShop) and blasticidin S (10 μg/mL, Sigma-Aldrich) for antibiotic selection. Cells were cultured for 2 weeks in the presence of puromycin and blasticidin S, and the clonal selection was performed afterward. Single cells were seeded in a 96-well plate and cultured for 3 weeks in 20% DMEM supplemented with puromycin (0.5 μg/mL) and blasticidin S (10 μg/mL). The clone with the highest ACE2 and TMPRSS2 expression was selected and propagated.

### 2.8. Immunofluorescence Assay

HeLa^ACE2^ cells were seeded on coverslips in 12-well plates (TPP Techno Plastic Products AG). After infection, cells were fixed using 4% formaldehyde, permeabilized with 0.5% Triton X-100, and non-specific binding sites were blocked by incubation with 10% bovine serum albumin (BSA; BioShop) in PBS overnight at 4 °C. Subsequently, cells were incubated for 2 h with 0.05 µg/mL mouse SARS-CoV-2 Nucleocapsid Protein Monoclonal Antibody (Bioss Antibodies, Woburn, MA, USA). Following incubation, cells were washed three times with PBS and incubated for 1 h with donkey anti-mouse antibody conjugated with Alexa Fluor 488 (2.5 µg/mL, Invitrogen, Warsaw, Poland), and phalloidin conjugated with Alexa Fluor 647 (0.2 U/mL, Invitrogen). Coverslips were washed twice with PBS, and DNA was stained with DAPI (0.1 μg/mL, Sigma-Aldrich) for 20 min at room temperature. Cells were washed with PBS before coverslips were mounted on glass slides in ProLong Diamond Antifade Mountant medium (Life Technologies, Warsaw, Poland) and sealed. Fluorescent images were acquired using a FLoid fluorescent microscope (Thermo Fisher Scientific) and processed using the ImageJ (ver. 1.52b) Fiji software package (Madison, WI, USA) [23].

### 2.9. Genomic DNA Isolation

Genomic DNA was isolated using the salt precipitation method described elsewhere [24]. Briefly, cells were scraped from flasks and lysed overnight at 55 °C in 1 mL lysis buffer (50 mM Tris, 50 mM EDTA, 1% SDS, pH 8) supplemented with 5 µL proteinase K (10 mg/mL; A&A Biotechnology, Gdańsk, Poland). Subsequently, RNase A was added to lysates at a final concentration of 10 µg/mL, and lysates were incubated for 30 min at 37 °C before being cooled on ice. A total of 667 µL of cold 7.5 M ammonium acetate (Sigma-Aldrich) was added before samples were thoroughly vortexed and centrifugated at 5000× *g* for 10 min. Supernatants were transferred to fresh tubes, and DNA was precipitated with 1 mL of isopropanol (BioShop). Samples were centrifuged at 5000× *g* for 10 min, and the resulting pellets were washed (5 min centrifugation at 5000× *g*) with 1 mL of 70% ethanol (BioShop). Air-dried pellets were resuspended in 50 µL of RNase/DNase-free water and incubated at 65 °C for 1 h, and then at room temperature overnight. gDNA concentrations were measured using Nanodrop 2000.

### 2.10. Preparation of NGS Library

Libraries were prepared by PCR amplification using gDNA as a template and primers corresponding to lentiviral sequences flanking the gRNA. PCR reactions were prepared as follows: 20 µL of 2× GreenTaq polymerase Mix, 1 µL each of PCR forward and reverse primer (10 µM), 100 ng of gDNA, and RNase/DNase-free water to a final volume of 40 µL. Tubes were placed in a thermocycler using the following program: 30 s at 95 °C, followed by 35 cycles of 30 s at 95 °C, 20 s at 54 °C, and 20 s at 72 °C; terminal elongation was performed for 7 min at 72 °C. Electrophoresis of amplified samples was performed for 40 min at 100 V with a 1.2% agarose gel. Bands of the expected size were excised from the gel and purified using the GeneJET Gel Extraction Kit (Thermo Fisher Scientific) and eluted in 30 µL of RNase/DNase-free water. The concentration of purified products was determined using the Qubit^®^ dsDNA HS Assay Kit (Thermo Fisher Scientific), as per the manufacturer’s instructions. Sequencing libraries were prepared using the Ion Plus Fragment Library Kit (Thermo Fisher Scientific), following the “amplicon libraries without fragmentation” protocol. Purified PCR product (100 ng) was added to end-repair reactions, and end-repaired amplicons were purified using Agencourt™ AMPure™ XP Reagent (1.8× sample volume; Beckman Coulter, Indianapolis, IN, USA). Barcoded adapters were ligated to amplicons using Ion Xpress™ Barcode Adapters 1 to 16 (Thermo Fisher Scientific). Barcode-ligated libraries were purified with the Agencourt™ AMPure™ XP Reagent (1.5× sample volume) and analyzed with a 2100 Bioanalyzer using the High Sensitivity DNA Assay (Agilent Technologies, Santa Clara, CA, USA).

### 2.11. Sequencing and Data Processing

The DNA concentration of libraries was normalized to 80 pM and pooled before template preparation using the Ion OneTouch™ 2 System (Ion PI™ Hi-Q™ OT2 200 Kit and Ion PI™ v3 chips; Thermo Fisher Scientific). Sequencing was performed on the Ion™ Torrent Proton System (Thermo Fisher Scientific) with 520 flows, which typically resulted in ~80 M single-end reads and sequencing depth of 1 M reads per sample, which was sufficient for our in-depth analysis.

Sequencing data in FASTQ format were further processed using a dedicated MAGeCK (Model-based Analysis of Genome-wide CRISPR-Cas9 Knock-out, accession date: 02.12.2020) tool [25]. The generated count table contains raw abundances for targeting and non-targeting gRNA from 48 samples (typically about 170 k counts per sample). Follow-up analyses were performed using the dedicated packages from Bioconductor (https://www.bioconductor.org/, version 3.10 [26]) in R (https://www.r-project.org/, [27]), accession date: 26 March 2021).

### 2.12. Between- and Within-Sample Normalization and Enrichment Score Calculations

Between- and within-sample normalization was performed using as a reference non-targeting controls with non-elevated expression, defined as belonging to the first mode of a typical multi-modal distribution. Per sample mean and standard deviation derived from these filtered populations of non-targeting controls were used for normalization, and these normalized abundances are referred to as “enrichment scores”.

### 2.13. Selection of Candidate Genes and Gene Set Enrichment Analysis

A rank-list was prepared from the sorted average enrichment scores. The cut-off was selected empirically by examining the agreement between lists of top genes of 16 samples (each ordered by individual sample enrichment). For this purpose, we have examined lists of top 100–200 genes and identified the local optimum, as in similar studies length of a list of genes of interest was from such range. A total of 178 genes of interest were identified for further in silico functional analysis and in vitro validation. PANTHER resources were used for the examination of gene function, while DAVID (https://david.ncifcrf.gov/summary.jsp, accession date: 28 April 2021 [28,29]), Metascape 3.5 (https://metascape.org/, accession date: 28 April 2021), and TopGO tools (R package version 2.38.1, [30]) were used for gene set enrichment analysis. The first two tools (DAVID and Metascape) were used to extensively analyze these gene sets and their co-expression network. It was done both on the level of Gene Ontology (GO), including examination of the biological process (BP), the cellular component (CC), and the molecular function (MF) of genes of interest, as well as on the level of the KEGG pathways. It was complemented by GO-based enrichment analysis with the use of the R package TopGO, where relation-based correction (Parent–Child) with Fisher exact test has been applied.

### 2.14. Gene Knock-Out by CRISPR/Cas9

gRNAs were designed for genes of interest identified by our high-throughput screen, with vectors prepared as described previously [22,31]. Briefly, sequences were cloned into the lentiCRISPRv2 vector (the plasmid was a gift from Feng Zhang; Addgene plasmid #52961) via BsmBI sites; amplified in Stbl3 *E. coli*; sequenced by the external services (Genomed, Warsaw, Poland); and transfected into HEK293T cells to produce lentiviruses as described above. Subsequently, lentiviruses were used to produce polyclonal HeLa^ACE2^ cells. Cells were selected in the presence of puromycin (0.5 µg/mL) for 2 weeks.

### 2.15. siRNA Silencing of Target Genes

A549^ACE2/TMPRSS2^ cells were seeded into 96-well plates (20,000 cells/well) and incubated for 24 h before the experiment. siRNA transfection was performed, both 24 h and 48 h later, to increase knock-down efficiency. Cells were transfected using RNAiMAX Lipofectamine (Thermo Fisher Scientific), and 1 pmol of ON-TARGETplus siRNA SMARTpool (ABO, Gdansk, Poland): EPGN (catalog ID: L-024183-02), USP17 (catalog ID: L-190093-00), MACF1 (catalog ID: L-013618-00), PCDHGA1 (catalog ID: L-013227-02), GAGE1 (catalog ID: L-011273-02), ACE2 (catalog ID: L-005755-00), or the Non-targeting Pool (catalog ID: L-019484-00). Reagents were mixed in 10 µL of OptiMEM (Thermo Fisher Scientific) per well, incubated for 5 min at room temperature, and added to cells dropwise.

### 2.16. Virus Replication Assay

For the assessment of viral replication, 10,000 HeLa^ACE2^ cells were seeded in each well of a 96-well plate (TPP Techno Plastic Products AG) and incubated for 48 h. Cells were infected with 50 µL of a mock-infection sample or purified virus (1000 TCID_50_/mL) in an infection medium (5% DMEM) for 2 h at 37 °C. Supernatants were discarded and cells were washed twice with 100 µL of 1× PBS before being overlaid with a 100 µL of fresh infection medium. Plates were incubated for 48 h at 37 °C, and virus-containing supernatants were collected and subjected to viral RNA isolation.

### 2.17. RNA Isolation

The Viral DNA/RNA Isolation Kit (A&A Biotechnology) was used for the isolation of viral RNA from cell culture supernatants and cellular RNA from cell lysates, according to the manufacturer’s instructions. Viral RNA was eluted in 30 µL of RNase/DNase-free water.

### 2.18. Reverse Transcription

Reverse transcription was carried out using the High-Capacity cDNA Reverse Transcription Kit (Life Technologies), according to the manufacturer’s instructions.

### 2.19. SYBR qPCR

cDNA yield was assessed using the GoTaq^®^ qPCR SYBR System (Promega, Pisz, Poland) and a real-time PCR thermocycler (CFX96 Touch Real-Time PCR Detection System, Bio-Rad, Warsaw, Poland) running the following program: 2 min at 95 °C, followed by 44 cycles of 3 s at 95 °C and 30 s at 60 °C (65 °C for USP17). cDNA was amplified in 10 µL reactions containing 5 µL of 2× GoTaq^®^ qPCR Master Mix and 1000 nM of each primer. The melting curve was analyzed for each pair of primers, with melting curve dissociation temperatures ranging from 60 °C to 95 °C. All data were normalized against GAPDH expression and presented as relative gene expression. The sequences of all primers used are listed in Supplementary Table S3.

### 2.20. RT-qPCR (Viral RNA Yield Analysis)

Viral RNA yield was assessed using the GoTaq^®^ 1-Step RT-qPCR System (Promega) and a real-time PCR thermocycler (CFX96 Touch Real-Time PCR Detection System, Bio-Rad) running the following program: 20 min at 45 °C and 2 min at 95 °C, followed by 40 cycles of 15 s at 95 °C and 1 min at 56 °C. RNA was reverse-transcribed and amplified in 10 µL reactions containing 5 µL of 2× GoTaq^®^ qPCR Master Mix; 0.2 µL GoScript™ RT Mix for 1-Step RT-qPCR; 200 nM probe labeled with 6-carboxyfluorescein (6-FAM) and Black Hole Quencher 1 (BHQ-1); and 600 or 800 nM of sense or antisense primer, respectively. The sequences of all oligonucleotides used are listed in Supplementary Table S3. To quantify nucleic acids, standards were prepared as described previously [32]. Eight 10-fold serial dilutions were used as a template to develop a standard curve.

### 2.21. Statistical Analysis of in Vitro Experiments

Data are presented as the mean ± SEM (standard error) from at least three independent experiments (biological repeats). Grubbs’ test (α = 0.05) was used to identify outliers. A *p*-value < 0.05 was considered statistically significant unless stated otherwise in the text.

## 3. Results and Discussion

### 3.1. CRISPR-Cas9 Genome-Wide Screening

**Figure 1:**
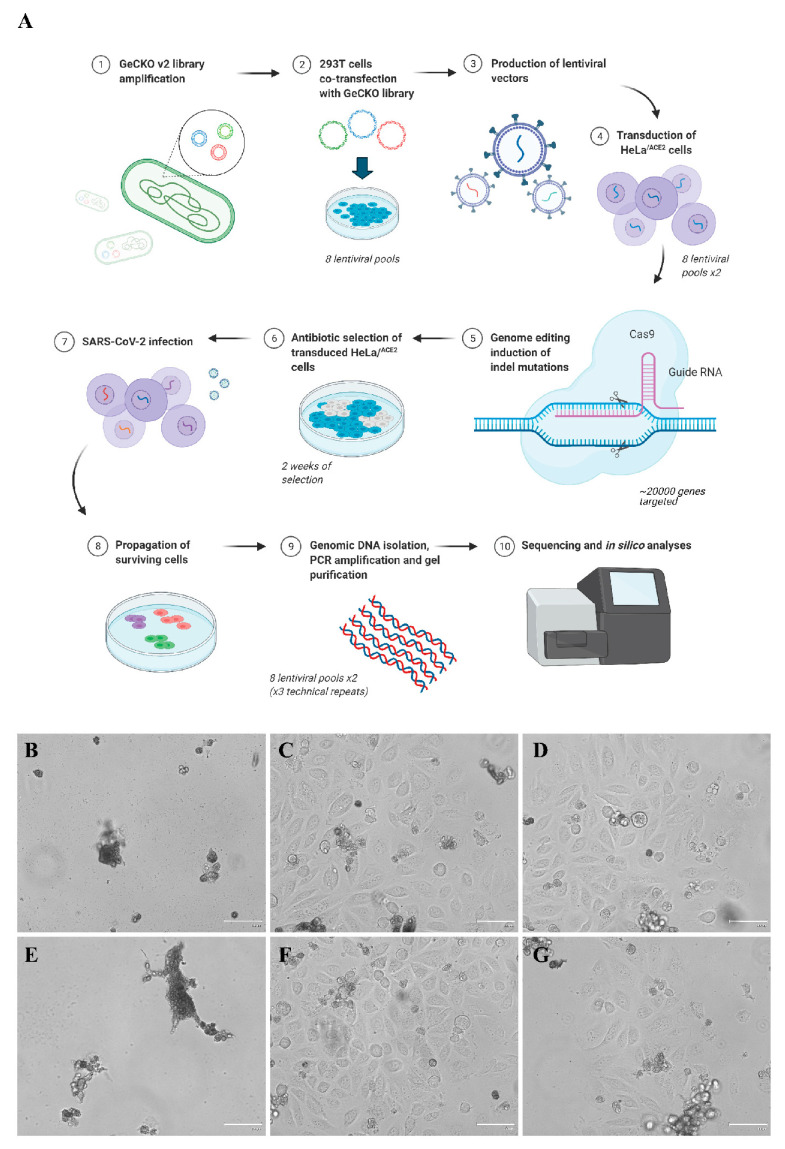
Overview of CRISPR/Cas9 genome-wide SARS-CoV-2 screening. The logic of the experimental setup. (**A**) SARS-CoV-2 infection of HeLa^ACE2^ cells harboring CRISPR knock-out library. Non-transduced HeLa^ACE2^ (**B**,**E**) and HeLa^ACE2^ GeCKO (transduced with CRISPR knock-out library pool) (**C**,**D**,**F**,**G**) cells were infected with SARS-CoV-2. Representative images were taken 7 dpi and present single-cell colonies surviving SARS-CoV-2 infection upon transduction with the library. Scale bar = 100 µm.

To identify cellular factors required for SARS-CoV-2 infection, we used a human CRISPR knock-out GeCKOv2 pooled library containing six gRNAs per gene (over 100,000 unique gRNAs in total), and 1000 non-targeting gRNAs to perform a genome-wide CRISPR-Cas9 screen in HeLa cells expressing the ACE2 entry receptor (HeLa^ACE2^), which is highly permissive to the SARS-CoV-2 infection resulting in CPE development and cell death [22]. We independently prepared eight lentiviral libraries (independent transfections and biological repeats) and transduced each library into two plates of HeLa^ACE2^ cells (two semi-biological repeats for each library). Three days post-transduction, the medium was supplemented with puromycin to select transduced cells. Following selection (7–10 days), both transduced and non-transduced cells were transferred to T75 flasks, grown to confluency, and infected with SARS-CoV-2 at 100,000 TCID_50_/mL. While no non-transduced cells survived the infection, some transduced cells started to recover and form colonies at 7 dpi (Figure 1A–B).

Cell clones that survived the infection were harvested, and their genomic DNA was isolated. As the lentiviral GeCKO vectors integrate with the genome, they may be PCR-amplified and sequenced. For every sample, PCR reaction was carried out thrice (technical repeats). After the amplification, PCR products were gel-purified and analyzed using the Ion™ Torrent Proton System. Approximately 61 × 10^6^ reads were generated using Ion PI™ Chip. Across the samples sequenced, the average read length was 146 bp (mean: 146 bp; median: 146 bp; range: 139–152 bp; standard deviation: 4 bp). The sequencing depth was typically 1 Mb (average reads per sample was 9.8 × 10^5^), which was sufficient for our analysis. We prepared and analyzed eight independent biological repeats (1°), for which two semi-biological repeats (2°) and three technical repeats (3°) were taken, resulting in 48 samples for analysis. This highly repetitive experimental design allowed us to perform high-quality and reliable analyses.

### 3.2. In Silico Analysis of Cellular Targets

**Figure 2:**
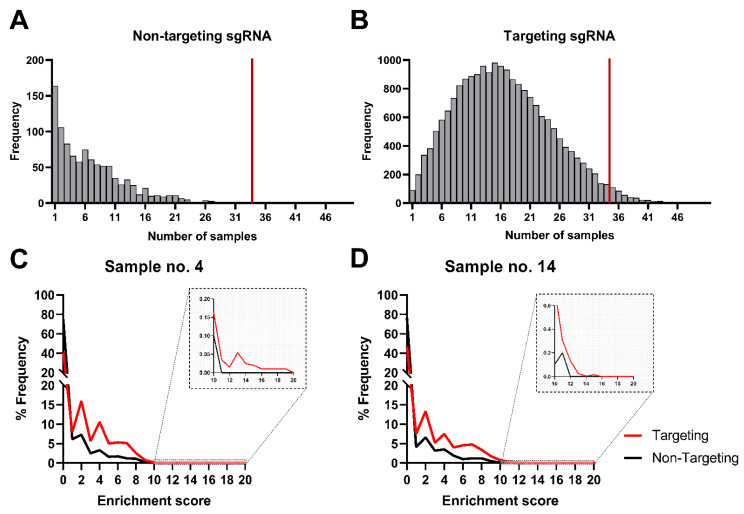
sgRNA frequency trends in non-targeting controls and targeting sgRNAs. (**A**,**B**) histograms present the distribution of sgRNA elevated abundance frequency (y-axis) among 48 samples (x-axis) for both non-targeting control and targeting guides, respectively. (**A**) summarizes the number of samples for which we detected control gRNAs, not targeting the human genome. The height of each bar corresponds to the number of non-targeting gRNAs that were detected. (**B**) A similar summary for gRNA targeting the genome is presented. The height of each bar corresponds to the number of gRNAs detected; however, the distribution differs. Panels (**C**) and (**D**) show density plots of normalized abundances (enrichment score) for two (from sixteen) representative samples for targeted (red) and non-targeted (black) sgRNA, respectively, which validates the performance of our normalization. The graphs from all 16 samples are presented in Supplementary Figure S3.

**Figure 3:**
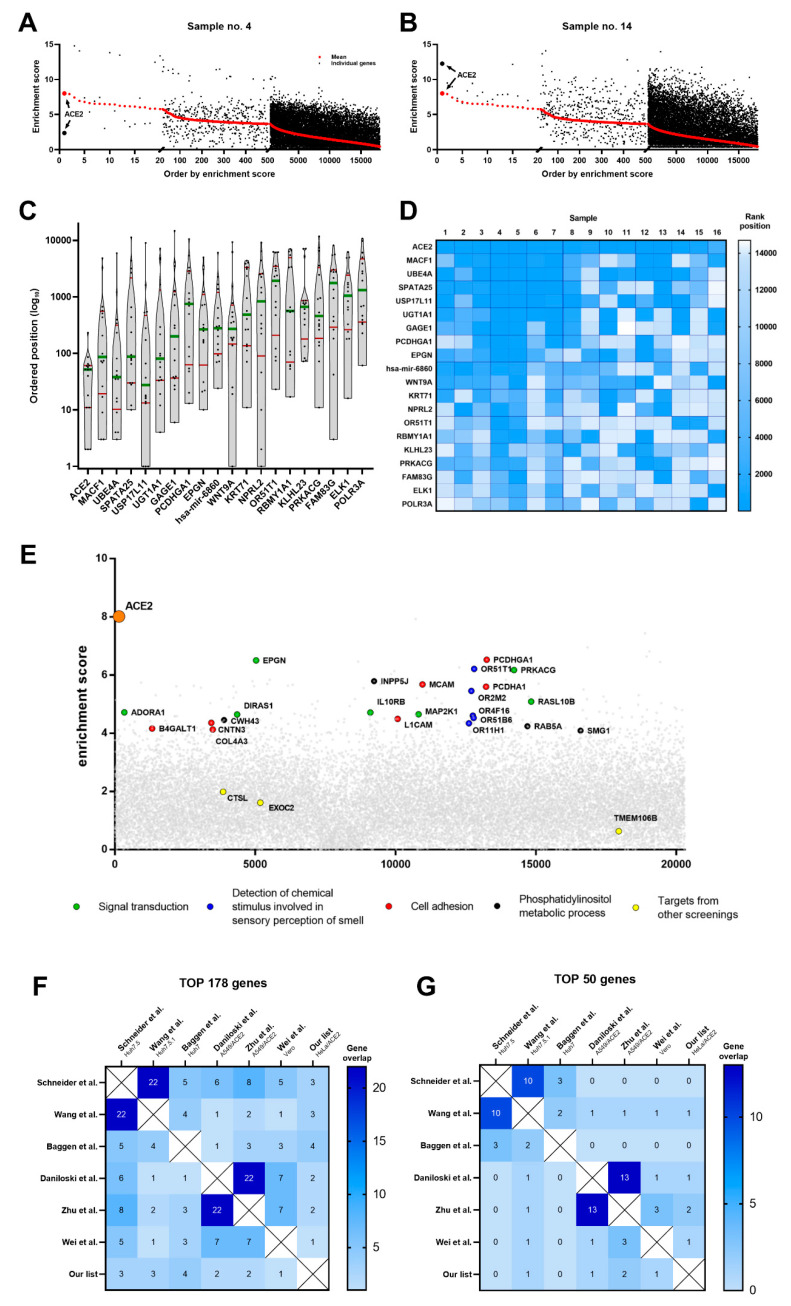
The selection of targets. (**A**,**B**) The distribution of sample-specific ranks genes. Bubble plots for gene distributions in two, representative samples, genes are ordered by mean enrichment score. (**C**) Violin plot for distribution of 20 top-ranked genes alongside the 16 biological repeats. Rank positions in single repeats were plotted, green line denotes median and red lines indicate interquartile range. (**D**) Heatmap for 20 top-ranked genes showing the rank position of individual samples. Top-ranked genes are ordered by the mean enrichment score. (**E**) Genome-wide CRISPR screening in HeLa^ACE2^ cells. Bubble plot of data from SARS-CoV-2 CRISPR screening, presenting distribution of genes in alphabetical order. Y-axis is presented as a mean of enrichment score from 16 independent screenings. (**F**,**G**) Heatmap for overlap of 178 and 50 top-ranked genes in our and other genome-wide CRISPR screenings.

Count tables were generated using the MAGeCK (Model-based Analysis of Genome-wide CRISPR-Cas9 Knockout) computational tool. For each sample, we obtained approximately 170,000 sequencing reads, which were analyzed in the R environment (https://www.R-project.org/). Within- and between-sample normalization was performed against the subset of non-targeting controls which typically do not show elevated levels (Figure 2A). The abundance of these non-targeting controls was elevated only in individual biological repeats, suggesting that they resulted from coincidental integration of random gRNA vectors into the host genome. In contrast, targeting gRNAs were observed in typically about 16, and in many cases 20 or more, biological and technical repeats (Figure 2B), confirming that the reads were not artifacts. Moreover, hundreds of targeting gRNAs were identified in 34 or more samples (threshold observed for non-targeting gRNAs—marked by the red vertical line on Figure 3A,B). These data indicate that, for targeting gRNAs, abundance elevation is non-random but biologically driven.

Next, we calculated the enrichment score for every gRNA against the normalized gRNA abundance. Normalization was carried out using the population mean and standard deviation calculated for selected non-targeting gRNAs for each of the 48 samples separately. We then have averaged those values among technical replicates to obtain a single set of enrichment scores for 16 individual biological samples. In each sample, targeting gRNAs were found to have greater enrichment scores than non-targeting gRNAs (Figure 2C,D and Supplementary Figure S3), validating our normalization. However, this enrichment score cannot be treated as a z-score per se because non-targeting controls and targeting gRNAs do not originate from the same population. The calculated enrichment scores represent observed biological signal strength and cannot be used to examine statistical significance. Moreover, non-targeting controls may lead to high false positives in growth-based screens if analyzed incorrectly [33].

To mitigate sources of biases, this study was designed with nested replicates; by calculating the average enrichment score for each gene from all 16 biological replicates, we were able to remove influence from confounding factors, which could cause significant variation. This is shown in Figure 3A,B, where the average enrichment score (red dots) is compared with the individual scores for the representative two samples (black dots). A considerable variation between enrichment scores between samples is observed, highlighting the relevance of the confounding factors.

From these average enrichment scores, a rank-list was prepared (Supplementary Table S1). The selection cut-off for candidate genes was estimated empirically by examining the agreement between top genes from all 16 samples. The local optimum was found for 178 genes, and those targets constituted our “top target” list.

The distribution of genes by sample-specific enrichment score displays a high degree of variation (Figure 3C,D), highlighting the need for nested replicate analyses to remove false positives. We, therefore, tested the validity of the design and analyses used in our nested replicate study by examining known factors required for infection in our dataset, such as the entry receptor ACE2. While ACE2 was not identified as the top hit in any of the 16 samples tested, it had the highest average enrichment score and was ranked 1st in our mean rank-list. To the authors’ knowledge is the first report showing such an extensive dataset for genome-wide screening during SARS-CoV-2 infection [9]. Additionally, only two other groups identified ACE2 as a top target [9,11], whereas other groups identified it on 4th, 8th, 77th, and 103rd positions [9,10,11,12]. This is concerning as ACE2 renders the cell susceptibility for SARS-CoV-2 infection, regardless of the applied model. TMEM106B was not identified in our screening, or in screens conducted in adenocarcinoma human alveolar basal epithelial cells (A549) and African green monkey cells (Vero). One may know that these hits may be highly model-dependent. Moreover, the protease cathepsin L (CTSL) was not identified as a hit in our screen, which was rather surprising as CTSL had been shown to play a role in viral entry during infection in vitro in cancerous cell lines [34]. One may hypothesize that other proteases in HeLa cells may be redundant, and therefore the cell will remain permissive despite lack of the particular enzyme. Consistent with other screens, we have not identified TMPRSS2, TMPRSS4, or neuropilin 1 (NRP1) as hits [35,36]. These factors have previously been shown to play a role in SARS-CoV-2 pathogenesis, ex vivo and in vivo; however, they have been shown to play little role in infection in vitro [37].

### 3.3. Pathway Analysis

Our experimental design has limitations resulting from applied state-of-the-art laboratory techniques that affect the execution and interpretation of downstream analyses. A typical expression-based enrichment analysis assumes that when the pathway is active, modulation of expression will occur for all or dominant fraction of pathway elements. The enrichment of active elements among all annotated is statistically tested. Here, this assumption is not valid; only some genes in a given pathway might display elevated abundance (defined through enrichment score) due to pathway redundancy and failure to silence genes which results from the characteristics/properties and limitations of the applied state-of-the-art laboratory techniques in our experimental design. This affects the interpretation of the data and although we were able to identify key genes from a given pathway, it was not possible to identify whole pathways with significance using state-of-the-art enrichment analysis approaches. On the other hand, using a certain model of infection will result in the identification of genes, and pathways specific for a particular model not necessarily universal for studied infection. To identify essential pathways involved in viral pathogenesis, we examined our top-ranked genes using the PANTHER computational tool. Among the top 178 most enriched genes, we identified seven genes involved in cell adhesion (*B4GALT1*, *CNTN3*, *COL4A3*, *L1CAM*, *MCAM*, *PCDHA1*, and *PCDHGA1*), four genes involved in phosphatidylinositol metabolism (*SMG1*, *CWH43*, *RAB5A*, and *INPP5J*), seven involved in signal transduction (*ADORA1*, *DIRAS1*, *EPGN*, *IL10RB*, *MAP2K1*, *PRKACG*, and *RASL10B*), and five genes required for the detection of chemical stimuli in the sensory perception of smell (*OR11H1*, *OR2M2*, *OR4F16*, *OR51B6*, and *OR51T1*; Figure 3E). Furthermore, we identified genes belonging to pathways that had previously been reported or suspected as having a role in SARS-CoV-2 infection: Golgi vesicle transport (*MACF1*); endosomal transport (*EVI5* and *RHOBTB3*); exocytosis (*JUP*, *CANT1*, *MVP*, and *SNX19*); regulation of signal transduction by the p53 class mediator (*TAF15*); response to oxygen levels (*ADORA1*); and epithelial cell differentiation (*NPHS2*, *JUP*, *ARHGEF26*, *KRTAP1-4*, *TGM3*, *TST*, *MAP2K1*, *KRT71*, and *SAFB2*; Supplementary Table S2). Interestingly, when we compared our data with data from previous studies, overlapping hits were only identified in studies using the same infection model (Figure 3F,G). On the other hand, some of the factors identified in the abovementioned screenings are known to play a role in the replication cycles of other viruses, what warrants further investigation [9]. It may be explained by the fact that in different cell lines multiple factors may play redundant roles, thus using the extended range of models will improve the understanding of the infection process. Nevertheless, this also supports our observation that hits identified in these screens are highly model-dependent and raises concerns about the possible over-interpretation of data. Some pathways strongly associated with infection were identified; however, these pathways, such as heparan sulfate proteoglycan biosynthetic processing, regulation of cellular pH, nucleosome disassembly, and Arp2/3 complex-mediated actin nucleation, were identified in only a few screens. We, therefore, modified our approach, examining pathways that appeared on all screens but with a lower overall score. Using this approach, we identified processes required for SARS-CoV-2 replication regardless of the model system. These processes included Golgi vesicle transport, endosomal transport, exocytosis, regulation of signal transduction by the p53 class mediator, response to oxygen levels, phosphatidylinositol metabolic processing, and epithelial cell differentiation.

Next, we performed functional enrichment analysis with the use of three complementary approaches (described Section 2), taking into account the aforementioned limitations (Supplementary Table S4). We did not find significant enrichment of pathways identified in previous genome-wide CRISPR screens, but we did identify pathways related to reproduction and cancer, including the developmental process required for reproduction (*ACE2, ADORA1, CTNNA3,* and *JUP*) or pathways associated with endometrial cancer (*CGB3*, *CAMK1D*, *NRPRL2*, *TXNRD3*, *JUP*, *GNB2*, *COL4A3*, *CDC16*, *MAD1L1*, *WNT9A*, *HLA-DQA1*, *PRKACG*, *CTNNA3*, *TCF7*, *MAP2K1*, and *ELK1*) but one may know whether it is possible that these findings are an artifact related to the cellular model used in this study, but it is also possible that these findings go some way to explain the complicated relationship between SARS-CoV-2 infection and the reproductive system [38].

While the virus strain and the type of the CRISPR/Cas gRNA library may affect the outcome of the screening, based on the lecture of the available literature one may conclude that the result depends strongly on the cellular model used. This discrepancy between the study may be confusing, but analysis of several models may allow for the identification of universal factors required for the virus replication that is common for all the models. However, to achieve that point other confounding factors need to be minimized. As we show in this work, the results obtained for a single analysis are unreliable and encompass non-specific hits. The method of analysis presented in this study allows for minimalization of the background noise and allows for a significant decrease of false results.

### 3.4. Validation of Top Hits

**Figure 4:**
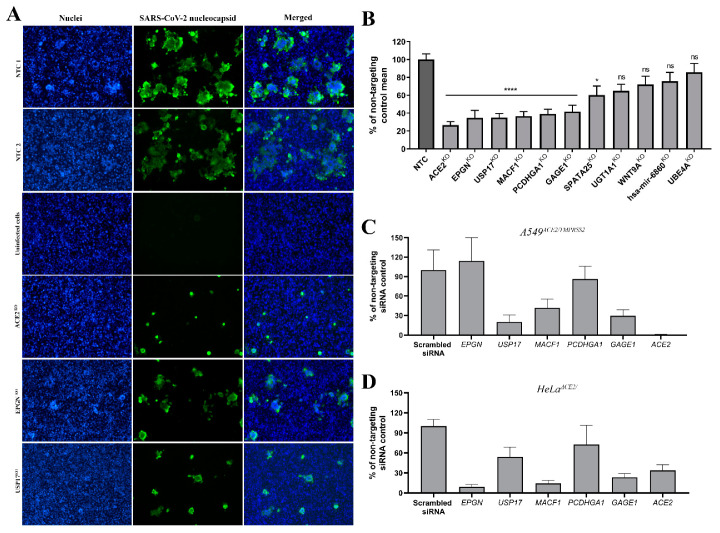
Identification of essential factors for SARS-CoV-2 replication in modified HeLa^ACE2^ and A549^ACE2/TMPRSS2^ cells. (**A**) Immunostaining assay was performed as described in Section 2; cells were fixed after 24 hours pi. Blue color denotes nuclei, green SARS-CoV-2 nucleocapsid protein. (**B**) SARS-CoV-2 replication was evaluated in modified HeLa^ACE2^ cells transduced with vectors harboring the template for sgRNA knockouts of *ACE2, EPGN, USP17, MACF1, PCDHGA1, GAGE1, SPATA25, UGT1A1, WTN9A*, *hsa-mir-6860*, and *UBE4A* genes. Two sgRNAs non-targeting any sequence in the genome were used as a control (NTC). Inhibition of viral infection was assessed 48 hours pi. by RT-qPCR, data were normalized and presented as percentage of the non-targeting control mean. Data are presented as a mean ± SEM from three independent experiments, each performed in triplicate or quadruplicate. Data were analyzed with Shapiro–Wilk and Brown-Forsythe tests. To determine the significance of differences between compared means, one-way ANOVA with post hoc Dunnett’s test was used. Values statistically significant are indicated by asterisks: * p < 0.05, **** p < 0.0001, ns—non-significant. SARS-CoV-2 replication was evaluated in modified A549^ACE2/TMPRSS2^ (**C**) and HeLa^ACE2^ (**D**) cells transfected with siRNA targeting *ACE2, EPGN, USP17, MACF1, PCDHGA1*, and *GAGE1* genes. Non-targeting siRNA was used as a control (NTC). Inhibition of viral infection was assessed 48 hpi by RT-qPCR, data were normalized and presented as percentage of the non-targeting siRNA control. Data are presented as a mean ± SEM from three independent experiments, each performed in triplicate or quadruplicate.

We used several approaches to validate our top hits. First, individual gRNAs with the highest number of reads were used to prepare single knock-out HeLa^ACE2^ cell lines (Supplementary Table S1), and the impact that these knock-outs had on SARS-CoV-2 replication was examined by RT-qPCR analysis (Figure 4B). One may remember that RT-qPCR method has some limitations such as sensitivity or restriction to detect only viral genetic material, and therefore we examined viral replication and spreading also by immunofluorescence (Figure 4B). Next, siRNA silencing was used to re-examine knock-outs significantly inhibited for viral replication in HeLa^ACE2^ and A549^ACE2/TMPRSS2^ cell lines (Figure 4C,D and Supplementary Figure S2). HeLa^ACE2^ cells are one of the accepted models for studying SARS-CoV-2 infection and pathogenesis [19]. While one may say that HeLa cells in such screening may have limitations and HeLa cells derived from the cervical cancer may not reflect the infection process in the healthy tissue, it is important to remember that all the in vitro models have their limitations, and that only by broadening the range of cells we may obtain the complete image. The A549^ACE2/TMPRSS2^ cell line was used for validation of results, as we believe that for several reasons it is superior to other models available. First, it is a human cell line, which supports the robust replication of SARS-CoV-2. Second, due to the presence of TMPRSS2, it recapitulates the natural route of entry for the coronavirus, which does enter via fusion directly on the cell surface. This allows the virus to be independent of the endocytic machinery and cellular cathepsins. Third, the A549 parental cell line was originally isolated from the lungs, which are the primary site for the replication of SARS-CoV-2 [39]. Unsurprisingly, gRNA targeting ACE2 reduced viral infection, and ACE2 was identified as our top hit. While knock-out of some top-ranked genes, such as *UGT1A1*, *WNT9A*, *hsa-mir-6860*, and *UBE4A*, resulted in only moderate decreases in viral replication, knock-out of *EPGN*, a gene that encodes epithelial mitogen, and epigen, a ligand for the epidermal growth factor receptor (EGFR) that plays a role in cell differentiation [40], conferred significant reduction in the number of viral RNA copies *(*Figure 4A,B). EGFR has previously been reported to play a role in SARS-CoV-2 infection [41]. However, we could not recapitulate these findings in A549^ACE2/TMPRSS2^ cells as they do not express EPGN (Figure 4C,D and Supplementary Figure S2) [42]. Knock-out of the deubiquitinase USP17, which affects cell proliferation and regulates inflammatory responses [43,44], similarly resulted in limited virus replication. Knock-down of USP17 resulted in a significant decrease in viral replication in both HeLa^ACE2^ and A549^ACE2/TMPRSS2^ cell lines (Figure 4C,D), in contrast to previous data [43]. USP17 is required for a variety of cellular processes, and a role for USP17 in SARS-CoV-2 infection may be difficult to interpret. Interestingly, both EPGN and USP17 may affect SARS-CoV-2 infection by an EGFR-associated pathway, as USP17 is required for the clathrin-dependent internalization of EGFR [45].

Further knock-outs, including *MACF1*, *PCDHGA1*, *GAGE1*, and *SPATA25*, resulted in a decrease of viral RNA copies compared to non-targeting control (NTC). Microtubule-actin crosslinking factor 1 (MACF1), also widely known as actin crosslinking factor 7 (ACF7), plays a role in various cellular processes, including the regulation of cell polarization and motility through an interaction with microtubules and F-actin. In the context of coronavirus infection, microtubules and actin are critical in the transportation of internalized virus-containing vesicles. Interestingly, ACF7 is also involved in a wide range of cellular signaling networks, including Wnt/β-catenin signaling, the upregulation of which is associated with inflammation and cytokine storm in COVID-19 patients [46,47]. SPATA25 (spermatogenesis-associated protein 25) may play a role in spermatogenesis; however, this role is poorly understood. GAGE1 is a poorly defined protein, and its role in SARS-CoV-2 infection is currently unknown. Further examination of our top 178 list of targets and literature, suggested two further genes for validation: B4GALT7 and IL10RB.

B4GALT7 is a galactosyltransferase that functions in the heparan sulfate biosynthesis pathway and plays a role in DENV viral replication. B4GALT7 was identified in our screen and others [11,12]. The IL10 subunit IL10RB was similarly identified from our top 178 list of targets. Protein levels of IL10 may influence COVID-19 disease outcomes [48]. We, therefore, tested whether the depletion of these genes has an impact on SARS-CoV-2 replication, and using RT-qPCR analysis we found that both *B4GALT7* and *IL10RB* KOs significantly reduced viral replication (Supplementary Figure S1), suggesting that they may play a role in SARS-CoV-2 infection. However, one may remember that using this methodology, only factors preventing productive infection would be identified.

We have prepared an extensive dataset using our genome-wide CRISPR/Cas9 knock-out screening approach, resulting in the identification of several cellular factors required for SARS-CoV-2 replication. This study was highly repetitive to remove false positives and stochastic noise, as the experiment design is very complex. We have identified and validated select factors using a broad range of analytic and experimental approaches. The data described provide an interesting insight into SARS-CoV-2 viral replication mechanisms and identify factors that may serve as potential therapeutic targets.

## Data Availability

Data is contained within the article or supplementary material.

## Supplementary Materials

The following are available online at https://www.mdpi.com/article/10.3390/cells10113159/s1, Figure S1: SARS-CoV-2 replication in modified HeLaACE2 cells, Figure S2: Analysis of siRNA gene silencing efficiency, Figure S3: Panels show density plots of normalized abundances for all sixteen samples for targeted (red) and non-targeted (black) sgRNAs, respectively, proving normalization performance and showing that for all cases there is a set of genes where enrichment score (x-axis) exceeds levels for non-targeting controls, Table S1: enrichment scores, Table S2: overlapping genes, Table S3: oligonucleotides sequences, Table S4: enrichment analysis.
