# Supplementary material for: Identification of Cellular Factors Required for SARS-CoV-2 Replication
Source: Cells. 2021 Nov 13;10(11):3159. doi: 10.3390/cells10113159 (PMC8622730; doi:10.3390/cells10113159)
Supplement: Supplementary file 1 [file cells-10-03159-s001.zip › Supplementary Figure S1.pdf]

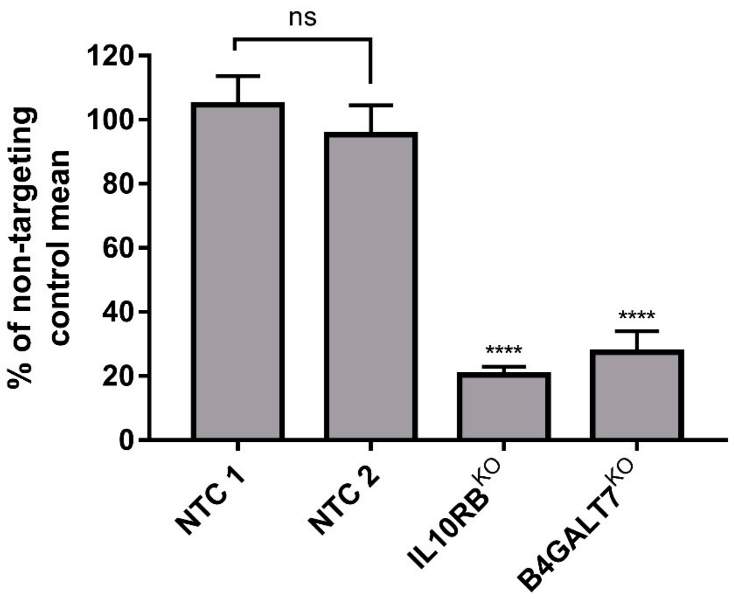

Supplementary Figure S1. SARS-CoV-2 replication in modified HeLa^{ACE2} cells. SARS-CoV-2 replication was evaluated in modified HeLa^{ACE2} cells transduced with vectors harboring the template for sgRNA knockouts of *IL10RB* and *B4GALT7* genes. Two sgRNAs non-targeting any sequence in the genome were used as a control (NTC). Inhibition of viral infection was assessed 48 hours p.i. by RT-qPCR, data were normalized and presented as % of the NTC number of viral RNA copies/ml. Data are presented as a mean ± SEM from three independent experiments, each performed in triplicate or quadruplicate. Data were analyzed with Shapiro-Wilk and Brown-Forsythe tests. To determine the significance of differences between compared groups, one-way ANOVA with post hoc Dunnett's test was used. Values statistically significant are indicated by asterisks: ****$p < 0.0001$.
