# Supplementary material for: Identification of Cellular Factors Required for SARS-CoV-2 Replication
Source: Cells. 2021 Nov 13;10(11):3159. doi: 10.3390/cells10113159 (PMC8622730; doi:10.3390/cells10113159)
Supplement: Supplementary file 1 [file cells-10-03159-s001.zip › Supplementary Figure S2.pdf]

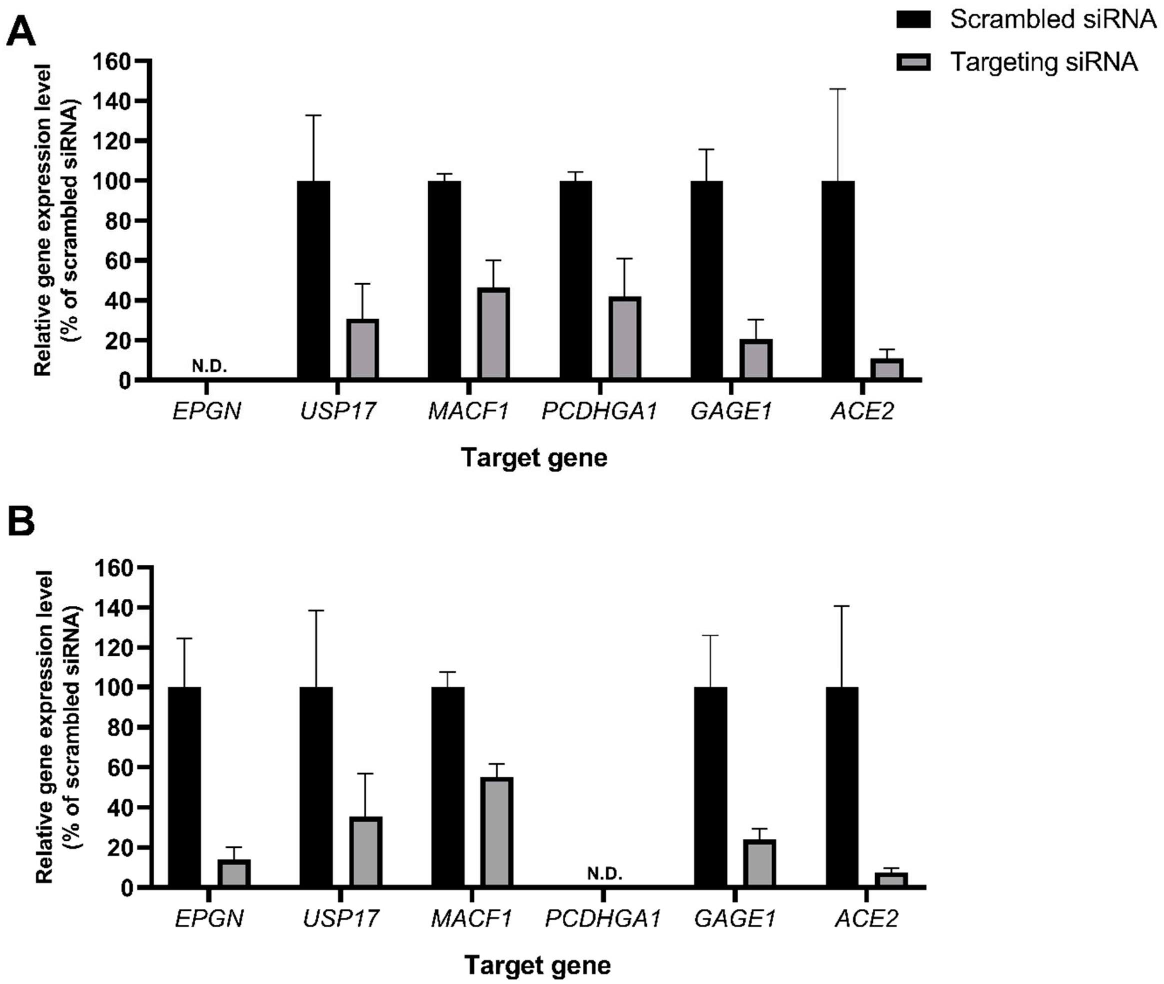

Supplementary Figure S2. Analysis of siRNA gene silencing efficiency. qPCR analysis of relative expression levels of EPGN, USP17, MACF1, PCDHGA1, GAGE1, and ACE2 in modified A549$^{ACE2/TMPRSS2}$ (**A**) and HeLa^{ACE2} (**B**). At 48 h following the first siRNA transfection, the cells were harvested and analyzed. RNA was isolated and subjected to RT-qPCR. Scrambled siRNA was used as a control. All data were normalized to GAPDH expression level and are presented as relative gene expression level (% of scrambled siRNA relative gene expression level). Data are presented as a mean ± SEM from two independent experiments performed in triplicates or quadruplicates. N.D. – Non detected.
