# Supplementary material for: Identification of Cellular Factors Required for SARS-CoV-2 Replication
Source: Cells. 2021 Nov 13;10(11):3159. doi: 10.3390/cells10113159 (PMC8622730; doi:10.3390/cells10113159)
Supplement: Supplementary file 1 [file cells-10-03159-s001.zip › Supplementary Figure S3.pdf]

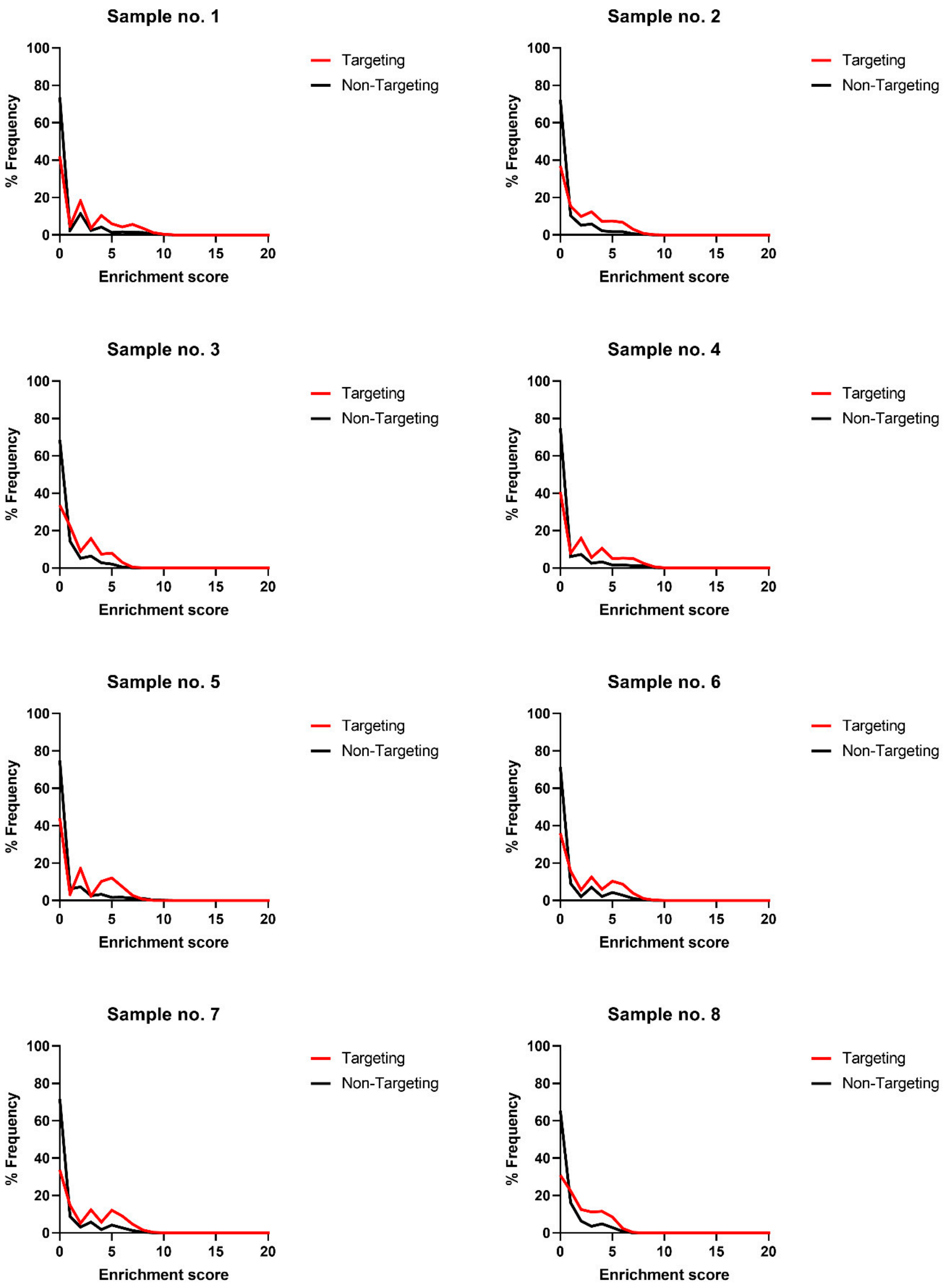

Sample no. 1
Sample no. 2
Sample no. 3
Sample no. 4
Sample no. 5
Sample no. 6
Sample no. 7
Sample no. 8
% Frequency
Enrichment score
0
20
40
60
80
100
5
10
15
Targeting
Non-Targeting

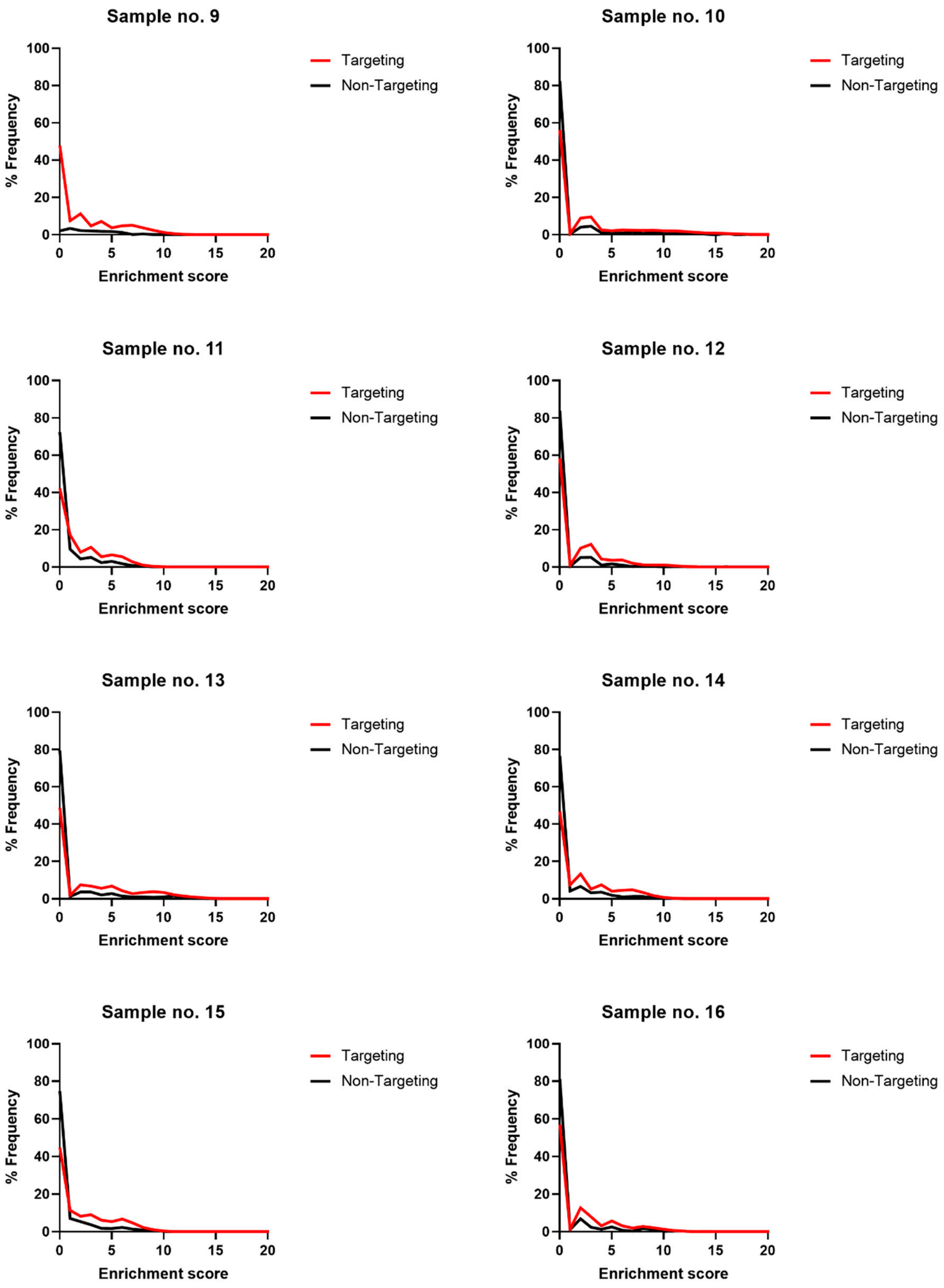

Supplementary Figure S1. Panels show density plots of normalized abundances for all sixteen samples for targeted (red) and non-targeted (black) sgRNAs, respectively, proving normalization performance and showing that for all cases there is a set of genes where enrichment score (x-axis) exceeds levels for non-targeting controls
